# Heterosis-Based Identification of Candidate Genes Associated with Lipid Metabolism and Meat Quality in Crossbred Pigs

**DOI:** 10.3390/ani16030423

**Published:** 2026-01-29

**Authors:** Teerath Kumar Suthar, Ziyi Zhao, Min Li, Jingbo Zhang, Yunpeng Zhang, Wu-Sheng Sun, Yuan Zhao, Shu-Min Zhang

**Affiliations:** 1 Key Laboratory of Animal Production, Product Quality and Security, Ministry of Education, College of Animal Science and Technology, Jilin Agricultural University, Changchun 130118, China; kumar@mails.jlau.edu.cn (T.K.S.); 18031975634@163.com (Z.Z.); 15931321811@163.com (M.L.); jingbo881003@live.cn (J.Z.); zhangyunpeng1218@163.com (Y.Z.); zhaoyuan4cl52@126.com (Y.Z.); 2 University of Chinese Academy of Sciences, Beijing 100049, China; 3 Jilin Provincial Engineering Research Center of Animal Probiotics, Jilin Provincial Key Laboratory of Animal Microecology and Healthy Breeding, Engineering Research Center of Microecological Vaccines (Drugs) for Major Animal Diseases, Ministry of Education, College of Veterinary Medicine, Jilin Agricultural University, 2888 Xincheng Street, Changchun 130118, China

**Keywords:** crossbred pigs, heterosis, intramuscular fat, meat quality, overdominance, transcriptomics

## Abstract

Heterosis or hybrid vigor is a well-recognized biological phenomenon, where crossbred progeny exhibit superior growth performance, stress tolerance, and productivity compared to their purebred parents. However, the molecular mechanisms underlying heterosis for meat quality traits in pigs remain poorly understood. In this study, we compared the longissimus dorsi muscle transcriptomes of Songliao Black Pig (SBP), Large White × Landrace pig (LWLDP), and their crossbred progeny (BXW) to uncover genetic mechanisms contributing to hybrid vigor in meat quality. Results identified 4290 differentially expressed genes (DEGs) in SBP vs. BXW and 3820 in LWLDP vs. BXW. Among these, 1358 genes displayed overdominant expression in BXW, indicating potential heterotic activation. Functional enrichment analysis identified key pathways involved in lipid metabolism, energy homeostasis, and muscle growth, including mTOR, AMPK, insulin, PI3K-Akt, MAPK, and calcium signaling. Over dominantly expressed genes such as *FASN*, *CPT1A*, *PPARG*, *ACACA*, *PPARA*, *SREBF1*, *FABP4*, *DGAT1*, *PPARGC1A*, *LIPC*, *ACSL5*, *PLCG2*, and members of the *COX family* were closely associated with intramuscular fat deposition, oxidative metabolism, and tenderness. Phenotypically, BXW exhibited improved meat color, moderate fat deposition, and lower shear force compared with parental breeds, clearly aligning with transcriptomic findings. These transcriptomic findings offer valuable insights into precision breeding strategies targeting superior meat quality through genomic selection and crossbreeding programs.

## Simple Summary

Heterosis, or hybrid vigor, refers to the improved traits seen in crossbred animals compared to their purebred parents. This study examined the muscle gene expression of Songliao Black Pig, Large White × Landrace pig, and their crossbred offspring to understand the genetic basis of better meat quality in hybrids. The crossbred pigs showed improved meat color, moderate fat content, and tenderness. Transcriptome analysis revealed key pathways and genes involved in fat metabolism and muscle development, such as mTOR, AMPK, and insulin signaling. These results help explain how crossbreeding improves meat quality and provide important insights for designing breeding programs focused on producing better-quality pork.

## 1. Introduction

Heterosis, or hybrid vigor, describes the enhanced performance of hybrid organisms in traits like stress tolerance, fertility, growth rate, and biomass production, when compared to their parent inbred lines [1]. This phenomenon is commonly utilized in breeding programs, where the crossing of two distinct purebred lines enhances the yield of both crops [2] and a similar mechanism works in livestock production [3]. There are three primary hypotheses that have been proposed to explain the genetic mechanisms underlying heterosis [4]: dominance [5], overdominance [6], and epistasis [7]. The advancements in functional genomics and RNA sequencing (RNA-seq) technologies have enabled the exploration of the molecular basis of heterosis by examining gene expression at the transcriptional level in various pig breeds [8,9].

With the continuous development of science and technology and production methods, people’s living standards and consumption levels are constantly improving, and the requirements for pork quality are also increasing. Hence, the production of high-quality and safe pork is becoming increasingly urgent [10]. Pork quality is influenced by many factors, including breed, nutrition, slaughter, and storage methods, with breed playing a crucial role [11]. In the past, the breeding goal was to increase the meat yield and reduce carcass fat. Large white pigs, Landrace pigs, and other lean pig breeds with the advantages of fast growth and high feed efficiency were cultivated. However, this also led to a significant decline in the quality of pork [4,12]. In contrast, indigenous pig breeds have the characteristics of good meat quality, strong stress resistance, and resistance to roughage, especially with a higher intramuscular fat (IMF), which is favored by consumers. The enhancement of meat quality traits in crossbred pigs through heterosis is increasingly recognized as a complex outcome of gene expression regulation, epigenetic modulation, and metabolic integration. One key molecular mechanism involves the upregulation of genes associated with muscle development and energy metabolism, particularly those governing oxidative muscle fibers and intramuscular fat (IMF) deposition, which are crucial for pork tenderness, juiciness, and flavor [13].

The present study aims to elucidate the molecular mechanisms underlying heterosis for meat quality by systematically comparing gene expression patterns in Longissimus dorsi muscles of pig crossbreds. Our goal was to identify candidate genes associated with improved meat quality through heterosis. These insights are predicted to facilitate genetic advancements in commercial pig populations, enabling more targeted breeding strategies aimed at enhancing meat quality. This, in turn, will address consumer demands and contribute to the economic growth of the pork industry.

## 2. Materials and Methods

### 2.1. Ethics Statement

The animal study protocol was approved by the Institutional Review Board (the Institutional Animal Care and Use Committee) of Jilin Agricultural University (20230609001 and 9 June 2023) for studies involving animals.

### 2.2. Animal Housing, Sex, and Feeding Management

In a previous study, we analyzed the fatty acids, amino acids, transcriptome, and metabolome data of Longissimus dorsi muscle to investigate the mechanism of fat deposition and meat quality in SBP and LWLDP [14]. Based on crossing of SBP and LWLDP, we reared fifty BXW (SBP × LWLDP), a crossbred line with a half-black and half-white coat pattern at Feimasi Animal Husbandry Co., Ltd., Gongzhuling National Agricultural Science and Technology Zone, Gongzhuling City, Jilin, China, for the identification of vigor’s characteristics of cross breeding in terms of meat quality. All pigs were raised under the same conditions and slaughtered using the same procedure as mentioned in the previous study. Furthermore, we assessed the similar meat quality traits along with L* at 24 h, a* at 24 h, b* at 24 h in BXW pigs, as mentioned in our previous study. Meat color was measured using the MiniScan EZ4500 spectrophotometer (HunterLab, Reston, VA, USA), with parameters including L* at 24 h, a* at 24 h, and b* at 24 h. After this, five pig samples from crossbreeding were randomly selected for transcriptomic comparison with our previous published SBP and LWLDP parent breed data, for identification of vigorous genes regulating meat quality that are transmitted from parents.

### 2.3. Library Construction, Sequencing, and Transcriptome Data Analysis

Five samples from BXW with two replications of each sample were used for sequencing. Library construction, sequencing, and transcriptomic data analysis was similarly followed with our previous study [14].

### 2.4. Heterosis Analysis and Classification of Expression Patterns

To assess the genetic basis of hybridization, mean expression of each gene in BXW was compared with both parental means. The mid-parent value (MPV) was defined as the average of SBP and LWLDP expression (MPV = SBP + LWLDP/2), while the higher parental value was designated as high parent. Mid-parent heterosis (MPH) and best parent heterosis (BPH) were analyzed through MPH = BXW − MPV/MPV × 100 and BPH = BXW − high parent/high parent × 100 formula. Genes were categorized into distinct expression patterns as follows: Transgressive Up (Overdominance): BXW significantly upregulated compared with both parents. Transgressive Down (Underdominance): BXW significantly downregulated compared with both parents. Genes with overdominance upregulation and positive BPH values were considered strong candidates contributing to hybrid vigor.

### 2.5. Identification of Hybrid Vigor Genes in the BXW Pig Crossbred

To identify candidate genes associated with hybrid vigor in the BXW crossbred, we performed transcriptomic comparisons between BXW and its parental lines, SBP and LWLDP, using RNA-seq data from the longissimus dorsi muscle. Differentially expressed genes (DEGs) were identified from two pairwise comparisons—SBP vs. BXW and LWLDP vs. BXW—based on *p*-value < 0.05, |log_2_FC| > 1. In this analysis, upregulated genes with higher expression in BXW relative to the corresponding parent were identified to detect transgressive expression patterns indicative of heterosis, we intersected the upregulated gene sets from both comparisons. Gene symbols were annotated using KEGG [15], and overlapping genes were prioritized as strong heterosis-related candidates. These were subsequently examined for functional relevance to muscle development, energy metabolism, and cellular stress responses.

### 2.6. Validation of DEGs

To validate the RNA-seq data, RT-qPCR was conducted on six selected genes (*DGAT1*, *PPARA*, *SREBF1*, *ACACA*, *PPARG*, and *CPT1A*). The RT-qPCR protocol followed the methodology described in previous studies [14,16]. The results were expressed as the mean and standard error, with the primer nucleotide sequences provided in (Table S1).

### 2.7. Statistical Analysis

Meat quality traits were compared across SBP, LWLDP, and BXW breeds using Tukey’s HSD post hoc test, which identified pairwise differences for significant traits. Superscript alphabets indicate statistical groupings; different alphabets denote significant differences between breeds. To explore the core regulatory genes associated with overdominance and underdominance expression patterns, protein–protein interaction (PPI) networks were constructed, and top 10 hub genes were identified using the string data base and Cytoscape v3.10.4. Pearson correlation coefficients(r) were calculated between meat quality trait and hub genes using Python 3.10v. Correlation results were visualized as a heatmap using seaborn, with statistically significant associations annotated with (*p* < 0.05 *, *p* < 0.01 **, *p* < 0.001 ***).

## 3. Results

### 3.1. Meat Quality Traits Among Parental and Hybrid Pigs

**Figure 1:**
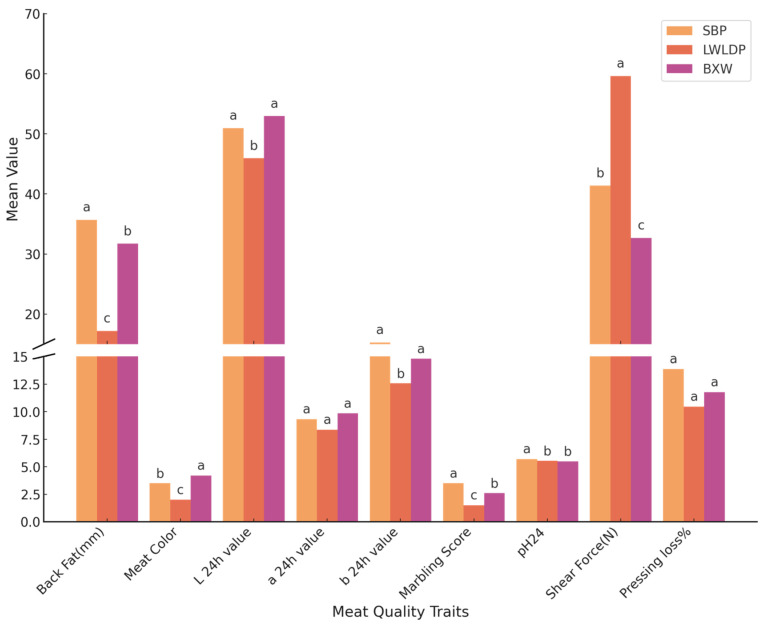
Comparison of meat quality traits among three pig breeds: SBP, LWLDP, and BXW. Data are presented as mean values for each trait. Bars represent breed-specific trait means, color-coded as SBP (orange), LWLDP (red), and BXW (purple). A broken *y*-axis was applied at 15 units to facilitate simultaneous visualization of traits with both high and low value ranges. Superscript letters (a, b, c) denote statistically significant differences between breeds for each trait (*p* < 0.05). The traits having same letter between breeds showing no significant difference.

Distinct variations in meat quality were observed among Songliao Black Pig (SBP), Large White × Landrace (LWLDP), and their hybrid (BXW) pigs (Figure 1). BXW exhibited intermediate backfat thickness (31.73 mm) between SBP (35.70 mm) and LWLDP (17.21 mm), reflecting partial dominance of adiposity. The hybrid showed superior meat color (score = 4.20) and greater lightness (L* = 52.96) relative to both parents, indicating improved visual quality. Marbling score followed the trend of SBP > BXW > LWLDP, suggesting inheritance of intramuscular fat traits from the indigenous breed. Notably, BXW displayed the lowest shear force (32.68 N), indicating enhanced tenderness and heterotic advantage. No significant differences were observed in pressing loss among breeds. While BXW demonstrated transgressive improvement in key sensory and textural attributes, it highlighted the phenotypic expression of hybrid vigor in meat quality.

### 3.2. Comparative Analysis of Transcriptomic Data of Three Pig Breeds

**Figure 2:**
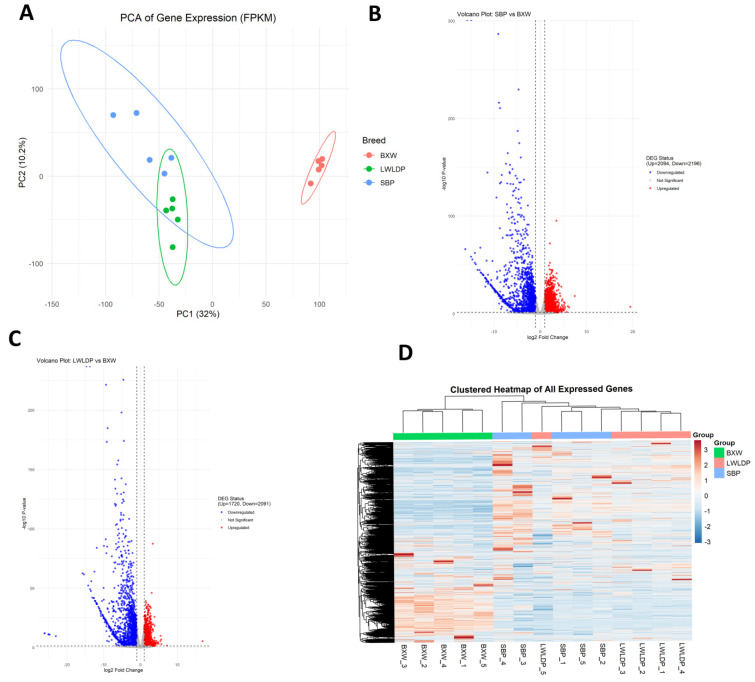
Transcriptomic analysis of gene expression in the longissimus dorsi muscle across three pig breeds (SBP, LWLDP, and BXW). (**A**) Principal component analysis (PCA) plot based on FPKM values showing transcriptome variance among BXW (red), LWLDP (green), and SBP (blue) breeds. PC1 (32%) and PC2 (10.2%) collectively explain the major variation in gene expression, demonstrating clear separation between groups. (**B**) Volcano plot of differentially expressed genes (DEGs) from the comparison between SBP and BXW. Genes with |log_2_ fold change| > 1 and adjusted *p* < 0.05 were considered significant. Red and blue dots represent up-regulated and down-regulated genes in BXW, respectively. (**C**) Volcano plot showing DEGs from the comparison between LWLDP and BXW, using the same significance criteria. Down-regulated (blue) and up-regulated (red) genes reflect differential expression patterns contributing to heterosis. (**D**) Hierarchical clustered heatmap of all expressed genes across the three breeds. Rows represent genes and columns represent individual biological replicates. Expression values are Z-score normalized (row-wise), and clustering reveals breed-specific transcriptomic profiles.

RNA-seq generated 39.8–68.0 million clean reads per sample across Songliao Black Pig (SBP), LWLDP, and crossbred BXW progeny. Mapping efficiency was high, with 91.9–98.2% of reads aligned to the reference genome, of which 94.0–95.5% was uniquely mapped. Most reads were assigned to annotated genes (94.6–97.5%) and exons (91.8–96.6%), with crossbred samples exhibiting slightly higher exon mapping proportions (Table S2). These results confirm the high quality of the transcriptomic data and its suitability for downstream analyses. A total of 21,280 genes were expressed in the samples of three breeds, with principal component analysis (PCA) of expressed genes within samples showing notable variation between breeds (Figure 2A; Table S3). Comparison DEG analysis between SBP vs. BXW identified 2196 down-regulated in BXW and 2094 up-regulated in SBP as shown in (Figure 2B), while comparison between LWLDP vs. BXW identified 2091 down-regulated and 1729 up-regulated in LWLDP as shown in (Figure 2C). The distribution of expressed genes in the sample of each breed is shown in (Figure 2D).

### 3.3. Identification of Over-Dominant Hybrid Vigor Gene and Functional Analysis

**Figure 3:**
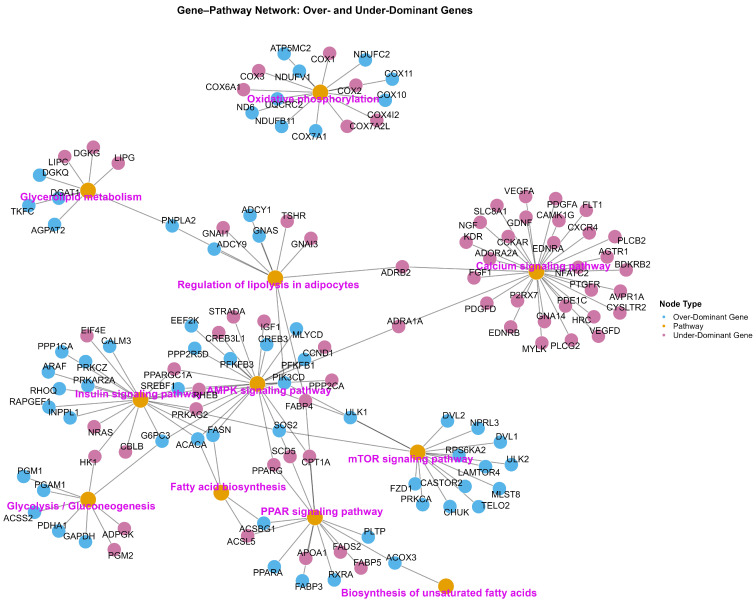
Gene–pathway network of over-dominant and under-dominant genes associated with lipid and energy metabolism and meat quality. This bipartite network illustrates the relationships between KEGG pathways (nodes in gold) and genes classified as over-dominant (blue) or under-dominant (red) based on heterosis-related expression patterns.

We identified 1358 over-dominant genes based on gene expression of BXW > SPB and LWLDP (Table S4) and 1291 under-dominant genes; BXW < SBP and LWLDP genes (Table S5). Functional enrichment analysis of over-dominant genes revealed several key biological pathways (Figure 3; Table S6). Among them the insulin signaling pathway includes *ACACA*, *FASN*, *SREBF1*, and *PIK3CD* genes, regulating lipogenesis, adipocyte differentiation, and glucose metabolism. The mTOR signaling pathway contains *RPS6KA2*, *ULK1*, *ULK2*, and *MLST8* genes that are regulating protein synthesis, cell growth, and skeletal muscle hypertrophy. In parallel, the AMPK signaling pathway contains *FASN*, *ACACA*, *G6PC3*, *PFKFB1*, and *CREB3* genes, reflecting active energy homeostasis and fatty acid oxidation. Additionally, the vascular smooth muscle contraction pathway was enriched with *ADCY1*, *ADORA2B*, and *PRKCA* involved in regulating vascular tone and muscle perfusion. Under-dominant genes with low expression in BXW compared with both parental breeds indicate transcriptional suppression following hybridization (Table S7). The KEGG enrichment analysis revealed that genes were primarily involved in immune regulation, cellular signaling, structural remodeling, and lipid metabolism. Downregulation of PI3K–Akt, MAPK, TGF-β, and Hippo signaling components (*CCND1*, *TGFB1*, *FOS*, and *BMP4*) indicates modulation of myogenic and extracellular matrix remodeling processes. Additionally, the focal adhesion and tight junction pathway gene (*ACTB*, *CDH5*, and *CLDN15*) imply transcriptional adjustments in cytoskeletal organization and tissue integrity. Overall, enrichment of lipid, atherosclerosis-related, sphingolipid, calcium, and cGMP–PKG signaling pathways point to hybrid-specific regulation of lipid metabolism, oxidative balance, and postmortem energy dynamics.

### 3.4. Hub Gene Identification

**Figure 4:**
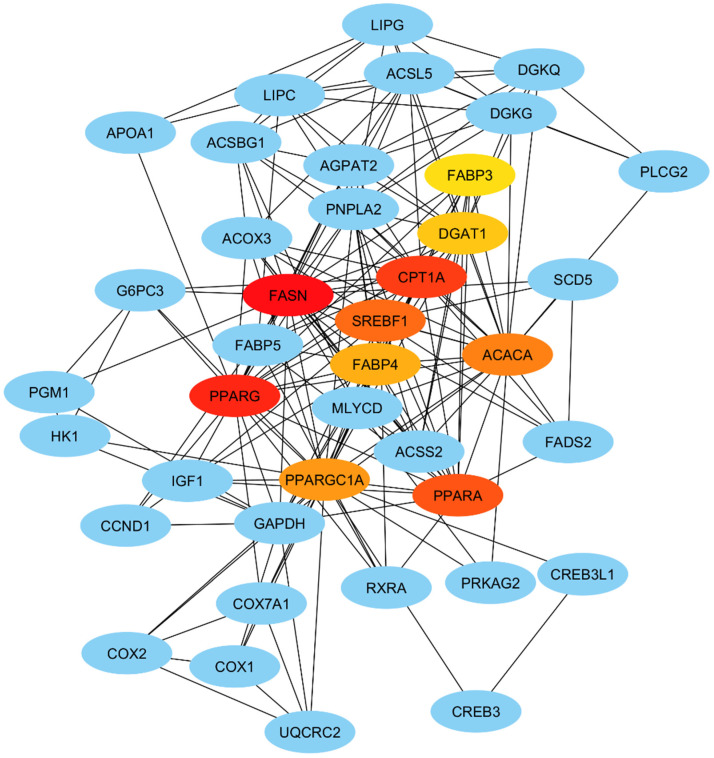
Gene interaction network of over-dominant lipid genes. Each node of the interaction network represents gene, and edges indicate known or predicted functional associations. The color of the nodes from red and oranges to light yellow represents the degree of connectivity from higher to lower, respectively. Red indicates hub genes (*FASN*, *CPT1A*), orange indicates moderately central genes (*SREBF1*, *ACACA*, *PPARA*), yellow to light yellow color genes (*FABP4*, *DGAT1*) indicate less degree of connectivity, and blue nodes represent first connectivity to hub genes.

We constructed a gene interaction network based on over-dominant and under-dominant genes that were selected from key pathways involved in the regulation of meat quality traits (Figure 4). The network comprises 63 genes interconnected by multiple interaction edges, highlighting the complexity of transcriptional and metabolic crosstalk underlying both lipid metabolism and meat quality regulation. Several hub genes, including *FASN*, *PPARA*, *SREBF1*, *ACACA*, *DGAT1*, and *FABP3* were over-dominant and *PPARG*, *CPT1A*, *FABP4* and *PPARGC1A* were under-dominant genes.

### 3.5. Pearson Correlation Between Hub Genes and Meat Quality Traits

**Figure 5:**
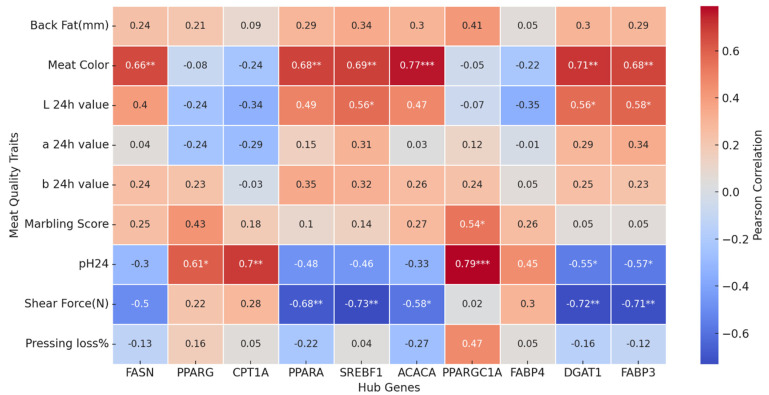
Heatmap illustrates the Pearson correlation coefficients between meat quality traits and hub genes. Color gradient indicates the direction and magnitude of correlations, with red indicating positive and blue indicating negative correlations. The intensity of the color corresponds to the strength of the correlation. Statistical significance is indicated by asterisks: *p* < 0.05 (*), *p* < 0.1 (**), and *p* < 0.001 (***). A scale bar is included to represent the range of correlation values.

Pearson correlation analysis reveals many statistically significant correlations (*p* < 0.05) between meat quality traits and hub genes (Figure 5). Among the positively correlated genes, *SREBF1* showed strong associations with meat color (r = 0.688, *p* < 0.01), and L value* (r = 0.560, *p* < 0.05), reflecting its role in lipogenesis and its potential influence on visual meat quality via intramuscular fat (IMF) deposition. Similarly, *ACACA* correlated positively with meat color (r = 0.765, *p* < 0.001), supporting its involvement in fatty acid synthesis. *PPARA*, a regulator of lipid oxidation, was also positively correlated with meat color (r = 0.680, *p* < 0.01) and L value* (r = 0.489, *p* < 0.05). Conversely, *CPT1A* exhibited negative correlations with L value* (r = −0.341, *p* < 0.05) and a value* (r = −0.289, *p* < 0.05), suggesting that increased fatty acid oxidation may contribute to color deterioration, possibly through enhanced oxidative stress. Importantly, shear force (N), an indicator of meat toughness, was negatively correlated with *SREBF1* (r = −0.731, *p* = 0.002) and *PPARA* (r = −0.680, *p* = 0.005), indicating that higher expression of these genes is associated with lower shear force and improved tenderness. *FASN* also showed a moderate negative correlation with shear force (r = −0.501, *p* = 0.057), consistent with the role of IMF in enhancing meat texture. These findings align with the established relationship where increased IMF contributes to greater tenderness by reducing connective tissue resistance and increasing juiciness.

### 3.6. RT-qPCR Validation

**Figure 6:**
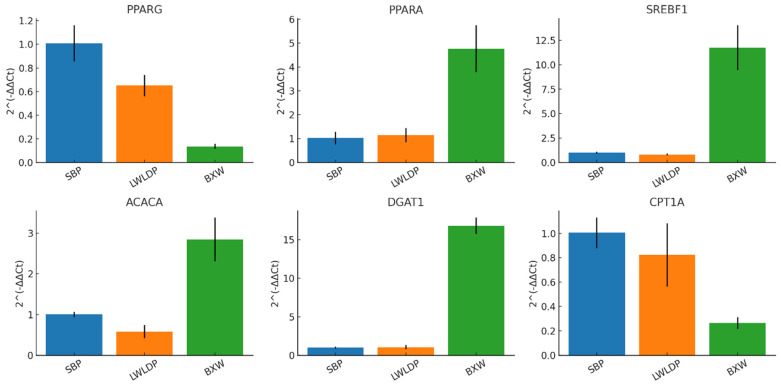
RT-qPCR validation of hub genes. Relative expression level verified by GAPDH (control) of the six selected DEGs in SBP, LWLDP, and BXW breeds.

To validate the RNA-seq results, six genes (*PPARG*, *PPARA*, *SREBF1*, *ACACA*, *DGAT1*, and *CPT1A*) were chosen for RT-qPCR analysis. The expression patterns observed in the RT-qPCR experiments closely matched those identified in the RNA-seq data (Figure 6). This consistency further validates the accuracy and dependability of the RNA-seq findings, confirming the ability of the transcriptomic approach to pinpoint crucial regulators of fat deposition and meat quality.

## 4. Discussion

The observed improvements in meat quality traits in the BXW crossbred pigs—such as enhanced tenderness, favorable meat color, and moderate intramuscular fat (IMF)—underscore the phenomenon of heterosis or hybrid vigor. BXW pigs inherited beneficial attributes from both parental lines: the high IMF and flavor traits of Songliao Black Pigs (SBPs), and the leanness and growth performance of Large White × Landrace pigs (LWLDPs). Notably, the BXW hybrids demonstrated transgressive segregation in traits like tenderness and meat color, exceeding both parental lines, thereby reinforcing the value of crossbreeding for meat quality enhancement.

At the phenotypic level, BXW exhibited significantly reduced shear force, a key indicator of tenderness, and higher L* values for meat color, aligning with consumer preferences. The intermediate backfat thickness and marbling scores in BXW reflect a partial dominance of fat deposition traits, suggesting effective transmission of IMF-related characteristics from SBP. Previous findings highlighted the advantages of indigenous breeds and the effects of crossbreeding. The SBP demonstrated greater backfat thickness, superior marbling, and higher intramuscular fat content compared to the LWLDP, reflecting the indigenous breed’s adiposity and flavor characteristics [14,17]. Such traits are important for meat palatability and consumer preference, as marbling positively correlates with juiciness and taste. The hybrid BXW showed intermediate backfat thickness and marbling, indicating partial dominance of fat-related traits inherited from SBP and LWLDP parental lines [18,19]. This aligns with the concept of heterosis, whereby crossbreeding improves certain phenotypic traits beyond the performance of either parent, as observed in the BXW transgressive improvement in meat color and tenderness. The improved meat color in BXW, evidenced by higher color scores and increased lightness (L*), suggests a favorable visual quality that can influence consumer purchasing decisions [20]. Notably, BXW displayed the lowest shear force values, indicating greater tenderness—an important sensory attribute—and validating the heterotic advantage of the crossbred. Shear force is inversely related to tenderness, and lower values confirm the improved texture in BXW meat. Pressing loss did not differ significantly among breeds, suggesting similar water-holding capacities in the muscles studied, supporting consistent juiciness among the groups [21]. In conclusion, the phenotypic expression of hybrid vigor in BXW demonstrates the potential of crossbreeding programs to enhance pork sensory traits, especially tenderness and visual appeal, which are critical to consumer acceptance and market value.

Functional analysis of over-dominant genes in the BXW hybrid pigs aims to enhance meat quality traits including insulin signaling AMPK and mTOR pathways. A previous study showed that these pathways contributes to fatty acid metabolism, intramuscular fat deposition, and skeletal muscle growth in Min pig [22]. Insulin signaling pathway (*ACACA*, *FASN*, *SREBF1*, and *PIK3CD*) plays a central role in lipogenesis, adipocyte differentiation, and glucose metabolism [23]. Their elevated expression in BXW indicates an enhanced capacity for lipid biosynthesis and energy utilization, which supports increased marbling and intramuscular fat deposition critical for meat flavor and juiciness. This pathway’s overdominance aligns with findings that improved fat metabolism is a hallmark of meat quality heterosis. Similarly, enrichment of the mTOR signaling pathway genes such as *RPS6KA2*, *ULK1*, *ULK2*, and *MLST8* is consistent with enhanced protein synthesis, cell growth, and muscle hypertrophy in the hybrid [24]. This hypertrophic response likely contributes to greater lean meat yield, a desirable trait combining growth performance with quality. Meanwhile, genes in the AMPK pathway such as *FASN*, *ACACA*, *G6PC3*, and *CREB3* reflect finely tuned energy homeostasis and fatty acid oxidation, indicating metabolic adaptability that supports efficient muscle growth and fat balance [25].

The enriched vascular smooth muscle contraction pathway genes (*ADCY1*, and *PRKCA*) influence vascular tone and muscle perfusion, factors that have been linked to postmortem meat tenderness and water-holding capacity—both crucial for consumer-acceptable meat texture and juiciness. *ADCY1* encodes an enzyme that catalyzes the conversion of ATP to cyclic AMP (cAMP), a pivotal second messenger in cellular signaling pathways. In muscle tissue, cAMP signaling influences muscle differentiation, hypertrophy, lipolysis, and metabolic regulation. Enhanced *ADCY1* activity may promote muscle growth and affect meat tenderness, marbling, and flavor development by modulating fat deposition and muscle cell differentiation [26]. *PRKCA* encodes an enzyme involved in various signaling pathways, including those regulating cell growth, differentiation, and apoptosis. In muscle tissue, *PRKCA* influences metabolic processes, muscle fiber type specification, and potentially the regulation of intracellular calcium, all of which can alter meat quality attributes such as texture, tenderness, and water-holding capacity [27,28]. These processes critically affect meat color and tenderness, reinforcing the muscle functional quality in BXW hybrids.

Our transcriptomic analysis revealed that genes such as *FASN*, *CPT1A*, *PPARG*, *ACACA*, *PPARA*, *SREBF1*, *FABP4*, *DGAT1*, *PPARGC1A*, *LIPC*, *ACSL5*, *PLCG2*, and members of the COX family were dominantly expressed in BXW. These genes are known to play pivotal roles in the regulation of lipid metabolism, oxidative capacity, and tissue remodeling, all of which are essential determinants of meat quality, including tenderness and color. The observed dominance of *FASN* (fatty acid synthase) in BXW suggests enhanced lipogenesis, which could explain the moderate fat deposition observed in the crossbred population. Fatty acid synthesis is a key process in adipocyte development and fat accumulation in the muscle tissue [29]. Additionally, *CPT1A* (carnitine palmitoyl transferase 1A) is crucial for mitochondrial fatty acid oxidation, and studies found that *CPT1A* knockdown (KD) promotes the differentiation of chicken preadipocytes into mature adipocytes [30]. This could contribute to improved meat color, which is often associated with higher oxidative muscle fibers and a more desirable muscle pH.

The upregulation of *PPARG* (peroxisome proliferator-activated receptor gamma), a master regulator of adipogenesis, along with *ACACA* (acetyl-CoA carboxylase), underscores the role of fatty acid metabolism in the development of intramuscular fat [31]. These genes are integral in managing energy storage and utilization within muscle tissue, which may explain the moderate fat deposition observed in BXW. Similarly, *SREBF1* (sterol regulatory element-binding transcription factor 1), which is involved in lipid biosynthesis, supports this fat deposition profile by promoting the synthesis of triglycerides and phospholipids in adipocytes [32]. Interestingly, regarding *PPARGC1A* (peroxisome proliferator-activated receptor gamma coactivator 1-alpha), a key regulator of mitochondrial biogenesis and oxidative metabolism, studies show that *PPARGC1A* polymorphism c.1288T > A is associated with pH and cooking loss in an F2 Duroc × Pietrain, coinciding with our results [32]. This suggests that enhanced lipid deposition, oxidative metabolism, and fatty acid composition could influence the mechanical properties of muscle fibers, making the meat more tender. Genes such as *FABP4* (fatty acid-binding protein 4), *DGAT1* (diacylglycerol O-acyltransferase 1), and *ACSL5* (long-chain acyl-CoA synthetase 5) are directly involved in lipid transport and synthesis, which are processes that contribute to the storage of intramuscular fat, which may affect the texture and tenderness of the meat [33,34,35]. Furthermore, *PLCG2* (phospholipase C gamma 2), a gene associated with cell signaling pathways, and the *COX* family (cyclooxygenase enzymes) are implicated in inflammatory responses and tissue remodeling, which could also influence muscle fiber structure and meat quality traits [36,37]. The modulation of these pathways might help explain the improved tenderness observed in BXW, as they may contribute to muscle fiber relaxation and reduced connective tissue formation. Collectively, the transcriptomic findings in this study align well with the phenotypic traits observed in BXW, including improved meat color, moderate fat deposition, and lower shear force. These results suggest that genetic modifications and gene expression changes in key metabolic and adipogenic pathways can have significant effects on meat quality traits. Our findings underscore the importance of a complex interplay between lipid metabolism, oxidative capacity, and tissue remodeling in determining meat tenderness and overall quality.

## 5. Conclusions

In conclusion, this study elucidates transcriptome-level regulatory patterns associated with heterosis in crossbred pigs, highlighting coordinated non-additive mechanisms relevant to meat quality formation. These findings support the hypothesis that hybrid vigor arises from system-level regulation of muscle development and metabolic homeostasis rather than isolated single-gene effects. The work provides a molecular rationale for integrating functional genomics into crossbreeding and selection strategies to improve pork quality and productivity. Further studies should validate key drivers and assess robustness across populations, environments, and multi-omics layers.

## Data Availability

The data of SBP and LWLDP were collected from our previous study under BioProject (ID: PRJNA1176353), and BXW breed raw data submitted to NCBI under BioProject (ID: PRJNA1394622).

## Supplementary Materials

The following supporting information can be downloaded at https://www.mdpi.com/article/10.3390/ani16030423/s1, Table S1: q-RT-PCR primers used for validation; Table S2: Summary of Transcriptomics; Table S3: FPKM gene expression values; Table S4: Identification of heterosis between parental and cross breeds; Table S5: Identification of over dominant and under dominant genes; Table S6: KEGG pathways analysis of over dominant genes; Table S7: KEGG pathways analysis of under dominant genes.

## References

[1] Birchler J.A., Auger D.L., Riddle N.C. (2003). In search of the molecular basis of heterosis. Plant Cell.

[2] Wei G., Tao Y., Liu G., Chen C., Luo R., Xia H., Gan Q., Zeng H., Lu Z., Han Y. (2009). A transcriptomic analysis of superhybrid rice LYP9 and its parents. Proc. Natl. Acad. Sci. USA.

[3] Hanot P., Herrel A., Guintard C., Cornette R. (2019). Unravelling the hybrid vigor in domestic equids: The effect of hybridization on bone shape variation and covariation. BMC Evol. Biol..

[4] Gao S., Nanaei H.A., Wei B., Wang Y., Wang X., Li Z., Dai X., Wang Z., Jiang Y., Shao J. (2020). Comparative transcriptome profiling analysis uncovers novel heterosis-related candidate genes associated with muscular endurance in mules. Animals.

[5] Bruce A. (1910). The Mendelian theory of heredity and the augmentation of vigor. Science.

[6] Shull G.H. (1908). The composition of a field of maize. J. Hered..

[7] Yu S., Li J., Xu C., Tan Y., Gao Y., Li X., Zhang Q., Maroof M.S. (1997). Importance of epistasis as the genetic basis of heterosis in an elite rice hybrid. Proc. Natl. Acad. Sci. USA.

[8] Ibanez-Escriche N., Varona L., Magallón E., Noguera J.L. (2014). Crossbreeding effects on pig growth and carcass traits from two Iberian strains. Animal.

[9] Haley C., d’Agaro E., Ellis M. (1992). Genetic components of growth and ultrasonic fat depth traits in Meishan and Large White pigs and their reciprocal crosses. Anim. Sci..

[10] Zhang S., Huang Y., Zheng C., Wang L., Zhou Y., Chen W., Duan Y., Shan T. (2024). Leucine improves the growth performance, carcass traits, and lipid nutritional quality of pork in Shaziling pigs. Meat Sci..

[11] Ramayo-Caldas Y., Mach N., Esteve-Codina A., Corominas J., Castelló A., Ballester M., Estellé J., Ibáñez-Escriche N., Fernández A.I., Pérez-Enciso M. (2012). Liver transcriptome profile in pigs with extreme phenotypes of intramuscular fatty acid composition. BMC Genom..

[12] Nevrkla P., Kapelański W., Václavková E., Hadaš Z., Cebulska A., Horký P. (2017). Meat quality and fatty acid profile of pork and backfat from an indigenous breed and a commercial hybrid of pigs. Ann. Anim. Sci..

[13] Zhao X., Hu H., Wang C., Wang Y., Lin H., Wang J. (2019). Transcriptome analysis of muscle reveals potential candidate genes and pathways affecting intramuscular fat content in Duroc pigs. Reaserch Sq..

[14] Kumar S.T., Zheng Y., Xu J., Zhao Z., Zhang Q., Zhang Y., Li M., Zou H., Azeem R.M., Sun W.-S. (2024). Transcriptome and metabolome insights into key genes regulating fat deposition and meat quality in pig breeds. Animals.

[15] Tang D., Chen M., Huang X., Zhang G., Zeng L., Zhang G., Wu S., Wang Y. (2023). SRplot: A free online platform for data visualization and graphing. PLoS ONE.

[16] Li J., Zhang D., Yin L., Li Z., Yu C., Du H., Jiang X., Yang C., Liu Y. (2022). Integration analysis of metabolome and transcriptome profiles revealed the age-dependent dynamic change in chicken meat. Food Res. Int..

[17] Almeida J., Bressan M., Santos-Silva J., Moreira O., Bettencourt C., Gama L. (2018). Physicochemical characteristics and sensory attributes of meat from heavy-weight Iberian and F1 Large White × Landrace pigs finished intensively or in free-range conditions. J. Anim. Sci..

[18] Liu H., He J., Yuan Z., Xie K., He Z., Zhou X., Wang M., He J. (2023). Metabolomics analysis provides novel insights into the difference in meat quality between different pig breeds. Foods.

[19] Chen Q., Chen Z., Sun Q., Zhang W., Wu F., Liu G., Wang T., Wang Z., Wang Q., Zhang J. (2024). Transcriptomic analysis of the longissimus thoracis muscle in pigs has identified molecular regulatory patterns associated with meat quality. Genomics.

[20] Li Y., He Y., Ran J., Huang Y., Li X., Jiang H., Li X., Pan Y., Zhao S., Song C. (2023). Comparison of meat quality and glycolysis potential of two hybrid pigs in three-way hybrid model. Front. Vet. Sci..

[21] Dan H., Liu C., Zhang H., Gan M., Wang Y., Chen L., Zhao Y., Liu B., Zhu K., Niu L. (2024). Integrated transcriptomic and metabolomic analyses reveal heterosis for meat quality of Neijiang pigs. Front. Vet. Sci..

[22] Liu Y., Yang X., Jing X., He X., Wang L., Liu Y., Liu D. (2017). Transcriptomics analysis on excellent meat quality traits of skeletal muscles of the Chinese indigenous min pig compared with the large white breed. Int. J. Mol. Sci..

[23] Czech M., Tencerova M., Pedersen D., Aouadi M. (2013). Insulin signalling mechanisms for triacylglycerol storage. Diabetologia.

[24] Pereira M.G., Dyar K.A., Nogara L., Solagna F., Marabita M., Baraldo M., Chemello F., Germinario E., Romanello V., Nolte H. (2017). Comparative analysis of muscle hypertrophy models reveals divergent gene transcription profiles and points to translational regulation of muscle growth through increased mTOR signaling. Front. Physiol..

[25] Chen J., Xiang J., Zhou M., Huang R., Zhang J., Cui Y., Jiang X., Li Y., Zhou R., Xin H. (2025). Dietary timing enhances exercise by modulating fat-muscle crosstalk via adipocyte AMPKα2 signaling. Cell Metab..

[26] Chen B., Zheng T., Bai X., Chang W., Zhang Y., Yang S., Li H., Li D., Wang T. (2025). The Metabolome in Different Sites of Gut Tract Regulates the Meat Quality of Longissimus Dorsi Muscle. Animals.

[27] Lv R., Li M., Xie R., Li Y., Li G., Shi J., Chen D., Luo H., Zhang Y., Zhou H. (2025). Transcriptomic and Metabolomic Profiling of the *Longissimus thoracis* Muscle Insights into Variations in Meat Quality between Hainan Black and Nubian Black Goats. LWT.

[28] Rodrigues L.F., Ramírez-Zamudio G.D., Pereira G.L., Torrecilhas J.A., Trevisan L.A., Machado Neto O.R., Chardulo L.A.L., Baldassini W.A., Curi R.A. (2024). Epigenetic insights into creep-feeding: Methylation profiling of Longissimus thoracis muscle at weaning in crossbred cattle. Front. Anim. Sci..

[29] Kim S.-W., Choi Y.-I., Choi J.-S., Kim J.-J., Choi B.-H., Kim T.-H., Kim K.-S. (2011). Porcine fatty acid synthase gene polymorphisms are associated with meat quality and fatty acid composition. Food Sci. Anim. Resour..

[30] Li Z., Li J., Lin Z., Zhang D., Zhang G., Ran J., Wang Y., Yin H., Liu Y. (2022). Knockdown of CPT1A induce chicken adipocyte differentiation to form lipid droplets. Braz. J. Poult. Sci..

[31] Puig-Oliveras A., Revilla M., Castelló A., Fernández A.I., Folch J.M., Ballester M. (2016). Expression-based GWAS identifies variants, gene interactions and key regulators affecting intramuscular fatty acid content and composition in porcine meat. Sci. Rep..

[32] Stachowiak M., Nowacka-Woszuk J., Szydlowski M., Switonski M. (2013). The ACACA and SREBF1 genes are promising markers for pig carcass and performance traits, but not for fatty acid content in the longissimus dorsi muscle and adipose tissue. Meat Sci..

[33] Nematbakhsh S., Pei Pei C., Selamat J., Nordin N., Idris L.H., Abdull Razis A.F. (2021). Molecular regulation of lipogenesis, adipogenesis and fat deposition in chicken. Genes.

[34] Jiang Y., Zhuang Z., Jia W., Xie M., Zhou Z., Tang J., Bai H., Chang G., Chen G., Hou S. (2022). Comparative Transcriptome Analysis Reveals the Key Genes Involved in Lipid Deposition in Pekin Ducks (*Anas platyrhynchos domesticus*). Agriculture.

[35] Xiao H., Zhao Z., Fang X., Yu H., Long X., Jiang P., Yang R. (2016). Association of the ACSL5 gene g. 33185918G > A and g. 33186348C > T mutations with carcass and meat quality traits of Chinese Simmental-cross steers. J. Anim. Plant Sci..

[36] Liu H., Xing K., Jiang Y., Liu Y., Wang C., Ding X. (2022). Using machine learning to identify biomarkers affecting fat deposition in pigs by integrating multisource transcriptome information. J. Agric. Food Chem..

[37] Fang C., Guo F., Zhao X., Zhang Z., Lu J., Pan H., Xu T., Li W., Yang M., Huang Y. (2022). Biological mechanisms of growth performance and meat quality in porcine muscle tissue. Anim. Biotechnol..

